# Change in attitudes after a suicide prevention media campaign in the Mid-Norway region

**DOI:** 10.1186/s12888-024-05905-x

**Published:** 2024-06-14

**Authors:** Christine Mohn, Egil Haga, Hanne Sofie Wernoe Nilsson, Jane Pirkis, Lars Mehlum

**Affiliations:** 1 Institute of Clinical Medicine, National Centre for Suicide Research and Prevention, University of Oslo, Sognsvannsveien 21, Oslo, 0372 Norway; 2 Norment Centre for Mental Disorders Research, Division of Mental Health and Addiction, Oslo University Hospital, Oslo, Norway; 3 Diakonhjemmet Hospital, Oslo, Norway; 4 Centre for Mental Health, University of Melbourne, Melbourne, Australia

**Keywords:** Help-seeking, Mental health stigma, Public campaign, Suicide, Suicide prevention

## Abstract

**Background:**

Suicide prevention media campaigns are one way of reaching people at increased suicide risk who would otherwise not seek help. This is the first study of a Norwegian campaign directed both at individuals at risk for suicide and at their social network.

**Methods:**

We evaluated a media campaign consisting of outdoor posters, feature articles, film clips, and online banners in print, digital, and social media spread across the Mid-Norway region in late autumn 2022. This campaign material consisted of information about how to seek help for suicide thoughts and mental health problems and how to help a friend in similar situations. Before and after this campaign, 1149 adult individuals living in Mid-Norway participated in a survey on attitudes to suicide, mental ill health, and help-seeking.

**Results:**

There were only marginal changes in attitudes and help-seeking literacy after the campaign. This result was sustained when controlling for age, sex, and campaign visibility. For males, there were a few changes in the negative direction, i.e. lack of willingness to seek help from family and friends, after the campaign.

**Conclusion:**

We conclude that the campaign did not seem to have the desired effect and suggest ways of improving future regional Norwegian media campaigns.

**Supplementary Information:**

The online version contains supplementary material available at 10.1186/s12888-024-05905-x.

## Introduction

Suicide rates in Norway have been largely stable the past three decades, with approximately 650 individuals (12.4 per 100 000) taking their own life annually [1]. Mental illness or psychological problems are strong predictors of suicide, with up to 90% of all individuals who take their own life having a psychiatric disorder [2]. However, over half of all individuals who die by suicide in Norway do not seek contact with specialist mental health services during the year prior to death [3]. Although improving treatment for inpatients and outpatients in health care services is an obvious and necessary way of reducing suicide rates, other measures must be taken to reach individuals at suicide risk who are not in contact with health care providers.

Suicide prevention media campaigns are one way of reaching people at increased suicide risk who would otherwise not seek help [4]. These campaigns often include help-seeking messages which directly target people at risk, and/or awareness-raising messages which indirectly increase help-seeking by equipping the general population to support people they may be concerned about. Both help-seeking and public awareness campaign messages typically consist of information about where to seek help for suicide thoughts (e.g., *Seek professional help. At helsenorge.no/selvmordstanker you will find advice about how to talk about suicide thoughts and where to find help*). Moreover, most campaigns contain elements intended to debunk myths about suicide (e.g., *Suicide happens without any warning signs*), as well as counter oversimplified or stigmatizing attitudes about suicide or mental illness (e.g., *People with depression are able to snap out of it*), or help-seeking (e.g., *Seeking help for emotional problems is a sign of weakness*). Belief in myths about suicide, prejudice or stigma towards people with mental illness, and poor general mental health literacy are powerful barriers to seeking professional help for mental health problems in general [5] and for depression and suicide thoughts in particular [6]. Targeting myths about mental illness and negative attitudes toward help-seeking aims to reduce suicide rates by helping at-risk individuals identify and change maladaptive thought patterns that may preclude them from seeking help. An equally important objective is to foster mental health literacy in the general population, increasing their readiness to and confidence in their ability to help a friend or family member who has suicide thoughts.

The success of previous awareness campaigns in reducing suicide rates and/or fostering mental health literacy has been limited. While attitudes to mental illness and help-seeking may improve after awareness campaigns in mass media, the effects on actual help-seeking and, ultimately, suicide rates are uncertain [7]. A major limitation is the typically low visibility rate of public campaigns, that is, the proportion of the targeted population that actually reports having noticed campaign messages in post-tests, with numbers as low as 15%. Nevertheless, those who do report having seen the campaigns may demonstrate more openness to seeking help for suicidal thoughts or mental illness compared to those who have not noticed them according to some studies [8, 9]. On the other hand, there have been some reports of more negative attitudes after public campaigns, with increases in taboos about suicide and lower estimation of the value of professional help after the intervention [8].

Demographically heterogeneous study samples may account for some of these conflicting findings. Male gender and older age have been linked to more negative attitudes to mental illness and help-seeking [10] as well as belief in suicide myths [11]. Moreover, the type of message (simple or complex) and the level of exposure, are other possible explanatory factors for the variability in findings [12]. Importantly, public awareness campaigns as a stand-alone measure to combat suicide and increase mental health literacy are likely to be insufficient [7, 12]. Suicide prevention media campaigns should probably be conducted while simultaneously strengthening and improving the availability of clinical services for the increasing number of individuals who may seek care after a campaign, and these treatment offers should be diversified and tailored to the needs of particular demographic sub-groups of people at suicide risk.

To achieve synergy effects between media campaigns, clinical services and other suicide preventive measures, WHO has recommended that these elements should be implemented in a coordinated way through so-called multi-level programs for suicide prevention [13]. The European Alliance Against Depression (EAAD) has developed and put to trial several multi-level programs in the last two decades. Ideally, the most effective program consists of four levels of intervention: (1) public awareness campaigns targeting the general public, (2) depression-treatment education for general practitioners (GPs), (3) education in depression recognition and assistance for help-seeking in community gatekeepers, such as pharmacists, teachers, police officers, religious leaders, and (4) establishing and supporting self-help groups for people with depression or for people who have attempted suicide and their families [13]. Norway has been a member of the EAAD network since 2016.

The present study uses data from a suicide prevention campaign that was conducted in the region of Mid-Norway, and which was the first of several regional campaigns in a Norwegian multi-level program for suicide prevention, which in turn was part of the current national strategy for suicide prevention [14]. The campaign was aimed both at awareness-raising and help-seeking. On the one hand, it aimed to improve mental health literacy in family members or friends of people at suicide risk, in order to strengthen their confidence in helping their loved ones. On the other hand, it sought to foster the willingness to seek help in people at increased suicide risk. Our research question was: After the campaign, what were the changes in people’s attitudes to helping others at suicide risk, and in people’s attitudes to seeking help for their own suicidal thoughts and mental health problems?

## Methods

### The “Talk about suicide thoughts – it may save lives” suicide prevention campaign

The Mid-Norway region includes two counties with 74 local municipalities and a population of approximately 700 000 spread across cities and rural areas, and was chosen as the campaign site. The population of this region was exposed to the campaign in weeks 43–50 of 2022. The campaign was instigated and organized by the Norwegian Directorate of Health (Helsedirektoratet). Major collaborating partners were the Regional Resource Centre for Suicide Prevention in Mid Norway (RVTS Midt) and the Mid-Norway Regional Health Authority (Helse Midt-Norge RHF), as well as local municipalities and regional NGOs. In the design and dissemination process of the campaign material, people with lived experience of suicide thoughts or attempts and people bereaved by suicide were involved as advisors.

Two elements of the campaign (the 1 min film clip and two interviews, see below) were published across Norway in its entirety. The same film material and rest of the campaign material was presented in the Mid-Norway region. The campaign had two main messages: *Talk about suicide thoughts – it may save lives* was chosen as the main and overarching message of the campaign as this wording is relevant for both people at risk and their social network. Additionally, the message *Stop.Ask.Listen. Are you worried about someone who has suicide thoughts* was directed at individuals close to someone who is struggling. The first message was presented on all campaign elements. The second message was added to some of the elements (see below). In addition to these main, short messages, longer descriptions of how to recognize suicide thoughts in oneself or others were included, as were messages that talking about suicide is safe and desirable if you are concerned about them, and that many people are willing to help. These were part of the campaign elements spread through online film clips and social media.

The public mass media material was designed by the Pol advertisement agency in cooperation with the Directorate of Health and the National Centre for Suicide Research and Prevention (NSSF). The communications agency Mindshare carried out the practical implementation of the campaign by placing the campaign content in print media and social media and on outdoor billboards. In addition, the RVTS Midt coordinated the non-paid organic sharing of the digital campaign material in social media. All campaign material included links or QR-codes to the campaign’s landing page at www.helsenorge.no, which is the central web page for health service information in Norway. At the www.helsenorge.no landing page, contact information for acute medical services, help lines, and support groups for people with suicide thoughts or mental illness were displayed. The campaign contained the following elements:

### Outdoor posters

During week 44, large, life sized posters were displayed at bus shed walls and billboard stands on pavements in the Mid-Norway regional capital Trondheim. The posters contained the message *Stop. Ask. Listen. Are you worried about someone who has suicide thoughts?.* Based on the number of travelers by bus and pedestrians assumed to pass these display points during the campaign period, this part of the campaign was intended to reach an estimated 171 000 individuals.

### Interviews in mainstream mass media

Two interviews were published with people with lived experience, one with a man who had survived a suicide attempt (‘Torbjørn’) and one with a woman who had lost her brother to suicide (‘Ann-Jorid’). The interview with Torbjørn was published in printed and digital local, regional, and daily newspapers. The interview with Ann-Jorid was published in national newspapers and weekly illustrated magazines. Moreover, one interview with Jakob Linhave from the Directorate of Health explaining about suicide thoughts and treatment was published in the same media channels. All interviews contained information from the Directorate of Health on how and where to seek help in a suicide crisis. The newspapers in which these interviews were published had a total of 420 000–470 000 readers, the numbers indicating the potential reach of this part of the campaign.

### Film clips

A film clip (1 min), and shorter versions (15 s) of this film clip, were published on social media channels (Facebook, Instagram, and Youtube) in the region. The film clip depicts a depressed man in different settings (waiting for the bus, working, having dinner with his family, attending a party) ending with a friend approaching him with a cup of coffee and the message *Talk about suicide thoughts – it may save lives* displayed across the screen. More than 700 000 individuals were exposed to different versions of the film during the campaign period in the region of all collaborating partners. The full version of the film was also shown in the commercial advertisements’ interval before the start of the feature films at cinemas all over Norway. Based on cinema ticket sales, a total of 600 000 individuals across Norway were exposed to the full version of the film in this way.

In addition, as the Mid-Norway region contains a sizable minority of Sami speaking individuals, two film clips (45 s each) depicting Sami middle aged men talking about stigma against suicide and mental illness in their community were published on Facebook and Instagram. This part of the campaign reached an estimated 165 000 individuals.

### Online banners

A smaller version of the outdoor poster, with the message *Stop.Ask.Listen*, was placed in social media channels (Facebook, Snapchat, and Youtube). Approximately 655 000 individuals were exposed to the campaign this way.

### Visibility and digital responses

To sum up, the entire adult population of the Mid-Norway region was indented to be exposed to campaign messages at least once during the campaign period. Mindshare, the bureau that implemented the campaign and measured its digital reach via number of subscribers and clicks, estimated that each adult was exposed to one or more campaign element(s) an average of 21 times. It should be noted that “being exposed” does not mean that the person actually noticed and read the content of the campaign element. The campaign generated approximately 31 000 clicks to the landing page at helsenorge.no from Mid-Norway. The click-through rate (CTR) was highest for Facebook and Instagram (0.36%) and lowest for Online banners (0.17%). Organic sharing in social media reached about 337 000 individual accounts.

### Participants

The participants in the current study were sampled by the Norstat company (which specializes in Norwegian population and marketing surveys) from their panel of respondents. Norstat has a fixed pool of possible respondents invited to participate in all surveys they administer. Inclusion criteria for participation are being aged 18 years or above and being able to fill in digital questionnaires in Norwegian on computers, tablets or smart phones. The sample used for the present study was stratified according to age, sex, and geographical location (ensuring representation from both regional counties) in order to achieve a balanced panel of respondents. See Table 1 for demographic characteristics.

**Table 1:** Demographic characteristics of the Mid-Norway sample pre campaign (*N* = 1149)

	Numbers / Mean (SD)	Test statistics
Sex	569 m, 580 f	X^2^ = 0.11, *p* 0.749
Age, years	51.5 (16.8), range 18–89	
Age group		X^2^ = 543.89, *p* < .001
< 30	151 (13.1%)	
30–39	167 (14.5%)	
40–49	203 (17.7%)	
50–89	628 (54.7%)	
Education level		X^2^ = 498.17, *p* < .001
Elementary school	36 (3.1%)	
Senior high school	226 (19.7%)	
Senior high school +	218 (19.0%)	
BA	338 (29.4%)	
MA	318 (27.7%)	
Other	13 (1.1%)	
Employment status		X^2^ = 358.91, *p* < .001
Public sector	311 (27.1%)	
Private sector	428 (37.2%)	
Not employed #	391 (34.0%)	
Other	19 (1.7%)	
Mean income level, personal *	5.7 (2.5), range 1–14	
Mean income level, household *	8.7 (3.8), range 1–16	
Number of individuals in household	2.2 (1.1)	
Number of children in household	1.4 (0.8)	
Living alone	275 (24.0%)	
Attempted suicide among family/friends	420 (36.6%)	
Suicide among family/friends	348 (30.3%)	

#: Among the not employed, 52.2% were retired, 27.5% were students, and 20.0% were disabled

*: Likert scale numbers of annual income, 1 = 100 000 NOK to 16 = 1 600 000 NOK

Participants were sent two surveys, the first in week 41 of 2022 (pre-campaign) and the second in week 3 of 2023 (post-campaign). The first digital invitation was issued to 7497 individuals of whom 1751 completed the pre-campaign survey, yielding a response rate of 23%. Among these, 1149 individuals participated in both the pre- and post-campaign surveys, giving a retention rate of 66%. This paper analyzes the responses of the 1149 individuals who completed both surveys. In this group, 265 (23.1%) stated that they had noticed a campaign on suicide awareness being carried out in the region, without having been prompted by examples of the campaign material.

### Survey

Through the survey, we obtained information on demographic, social, and economic characteristics in addition to the participants’ previous experience of suicide or suicide attempts among family and friends. Moreover, the following items assessed attitudes towards suicide and mental illness (Table 2): One item was taken from the Depression Stigma Scale (DSS) [15] where responses were made on a five-point Likert scale from 1 (“Completely disagree”) to 5 (“Completely agree”). Six items were taken from the Attitude to Suicide Scale (ATTS) [16] where responses were made on a five-point Likert scale from 1 (“Completely disagree”) to 5 (“Completely agree”). Three items were taken from the Attitudes Toward Seeking Professional Help scale (ATSPH) [17] where response were made on a four-point Likert scale from 1 (“Disagree”) to 4 (“Agree”). Five items were taken from the Self-stigma of Seeking Help scale (SSOSH) [18] with responses made on a five-point Likert scale from 1 (“Completely disagree”) to 5 (“Completely agree”). Four items were taken from the Multidimensional Scale of Perceived Social Support (MSPSC) [19] with response made on a five-point Likert scale from 1 (“Completely disagree”) to 5 (“Completely agree”). In addition, we constructed two items on confidence in getting good health care and feeling OK about seeking help from family and friends if experiencing emotional problems, both with responses made on a five-point Likert scale from 1 (“Completely disagree”) to 5 (“Completely agree”). Two items were questions about the history of suicide or suicide attempts among family and friends, where answers were given as “Yes” or “No”. Finally, two questions asked the respondents what they would do if they were worried about a friend who had suicide thoughts, and where they would seek help if they experienced suicide thoughts themselves. Several possibilities for action were listed as alternatives, and the respondents answered “Yes” or “No” to each (Tables 2 and 3).

**Table 2:** Attitudes to suicide and help-seeking pre and post campaign in Mid-Norway (*n* = 1149)

	Pre Mean (SD)	Post Mean (SD)	F-test
**Attitudes to suicide**			
I am prepared to help a suicidal person by contacting/talking to him/her	4.2 (1.0)	4.1 (1.0)	0.51 *p* = .477
When a person has decided to take their life, it can’t be prevented	2.1 (1.0)	2.1 (1.0)	1.57 *p* = .210
Suicide is one’s own business that others should not interfere with	1.6 (0.8)	1.6 (0.8)	1.87 *p* = .172
There is a risk of evoking suicide thoughts if one asks about it	2.7 (1.0)	2.6 (1.0)	0.40 *p* = .842
Suicide is a topic that should not be discussed	1.7 (0.8)	1.7 (0.9)	0.10 *p* = .748
Suicide occurs without warning	3.0 (1.1)	3.0 (1.1)	0.02 *p* = .884
If I had suicide thoughts, I am confident of getting good health care #	3.0 (1.1)	3.1 (1.1)	3.66 *p* = .056
**Attitudes to help seeking**			
If I had depression, I would not tell anyone	2.8 (1.1)	2.8 (1.1)	1.94 *p* = .164
If I were experiencing a serious emotional crisis, I am confident that I would get good professional help	2.6 (0.9)	2.7 (0.9)	1.01 *p* = .316
People should work out their own problems, getting psychological help should be the last resort	1.5 (0.7)	1.5 (0.7)	1.10 *p* = .295
Emotional problems, like many things, tend to work out by themselves	2.4 (0.8)	2.4 (0.8)	3.39 *p* = .066
**Self-stigma towards help seeking**			
If I sought professional help I would be less satisfied with myself	2.1 (1.0)	2.2 (1.1)	0.26 *p* = .610
My self-esteem would NOT be threatened if I sought professional help	3.9 (1.1)	3.9 (1.1)	0.02 *p* = .900
I would feel inferior if I asked a mental help counselor for help	2.1 (1.1)	2.2 (1.1)	2.67 *p* = .103
I would have negative feelings for myself if I couldn’t solve my own problems	2.8 (1.1)	2.7 (1.1)	0.01 *p* = .954
I would feel OK deciding to seek out professional help	4.1 (1.0)	4.0 (1.0)	0.20 *p* = .654
**Social support**			
If I needed it, I would feel OK seeking help from family and friends for emotional problems #	3.7 (1.0)	3.7 (1.0)	0.17 *p* = .691
I get the emotional support I need from my family	3.6 (1.1)	3.6 (1.1)	0.03 *p* = .868
I can trust my friends when something goes wrong	3.8 (1.0)	3.7 (1.0)	0.27 *p* = .607
I can talk to my family about my problems	3.6 (1.1)	3.6 (1.0)	2.03 *p* = .155
I can talk to my friends about my problems	3.6 (1.0)	3.6 (1.1)	0.92 *p* = .338

Repeated measures ANOVA with age group as covariate. #: Items formulated by us. See Methods section for information of the origin of the other items

**Table 3:** Attitudes to help-seeking alternatives pre and post campaign in Mid-Norway (*n* = 1149)

	Pre *N* (%)	Post *N* (%)	X^2^ -test
**What would you do if you were worried about a suicidal friend?**			
I would talk to that person about it	703 (61.2)	707 (61.5)	0.03 *p* = .869
I would ask him/her to contact professional help	583 (50.7)	582 (50.7)	0.00 *p* = 1.000
I would contact his/her next-of-kin	529 (46.0)	472 (41.1)	8.06 *p* = .005 **
I would contact health care services myself	384 (33.4)	359 (31.2)	1.94 *p* = .164
I am not sure what I would have done	167 (14.5)	185 (16.1)	1.61 *p* = .205
**If you had suicide thoughts, where would you seek help?**			
Family/partner/spouse/significant other	472 (41.1)	465 (40.5)	0.12 *p* = .729
Friends/colleagues	227 (19.8)	221 (19.2)	0.11 *p* = .746
GP	466 (40.6)	507 (44.1)	5.11 *p* = .024 *
Emergency services	136 (11.8)	146 (12.7)	0.55 *p* = .459
Local mental health services	294 (25.6)	318 (27.7)	1.90 *p* = .168
Telephone help line /emergency hotline	346 (30.1)	342 (29.8)	0.03 *p* = .862
Internet	143 (12.4)	158 (13.8)	1.03 *p* = .311
I would not seek help or information	83 (7.2)	81 (7.0)	0.01 *p* = .910
I don’t know where I would seek help	162 (14.1)	141 (12.3)	2.58 *p* = .108

Two-related samples McNemar test. **: *p* < .01, *: *p* < .05

The post-campaign survey contained additional questions about whether the respondents had noticed one or more of the campaign elements, and, if they had, what their impression of those elements were.

### Ethical approval

All respondents had given their broad, informed consent to membership of the Norstat web panel. This broad consent implies the right to refrain from participation in surveys without having to state a reason for declining. This project was carried out in keeping with the principles of the Helsinki Declaration.

### Statistics

#### Main analyses

The continuous variables among the pre-post survey responses were analyzed with repeated measures ANOVA with age group entered as a covariate (Table 2). Age group was chosen as covariate due to this factor being associated with mental health literacy and suicide myths [10, 11]. The dichotomous variables among the pre-post survey responses were analyzed with the Two-related samples McNemar test, where each person acts as their own control, eliminating the need for covariate control (Table 3).

#### Subgroup analyses

Using Independent samples t-tests and Mann-Whitney U-tests, we analyzed the male respondents only, as male gender has been linked to mental health stigma and belief in suicide myths [10, 11]. Then, we analyzed the pre-post responses of the proportion of the participants (*n* = 265, 23%) who in the post-test reported that they had noticed the campaign (without being shown any campaign material) using Paired samples t-tests and McNemar tests (Supplementary Tables 1–2). Finally, we compared the baseline responses of the 1149 (66%) individuals who had completed both the pre and the post campaign surveys with the attrition group of 602 individuals (34%) who were lost to follow-up, using Independent samples t-tests and Mann-Whitney U-tests (Supplementary Text).

All statistical analyses were performed with IBM SPSS version 29.

## Results

### Main analysis

The results of the main (*N* = 1149) repeated measures ANOVAs with age group as covariate showed no statistically significant changes in attitudes to suicide/suicidal people, mental illness, or help-seeking after the awareness campaign. (Table 2). According to the McNemar tests of dichotomous responses, after the campaign, a significantly lower proportion of respondents would contact the person’s next-of-kin if they were worried about someone with suicide thoughts, and significantly more people would contact their GP if having suicide thoughts themselves (Table 3).

### Subgroup analysis: males

When analyzing men separately from pre- to post-campaign (*n* = 569), there was a statistically significant decrease in their stating “I can trust my friends when something goes wrong” (mean 3.8 [SD 1.0] versus 3.7 [SD 1.0], t = 2.37, *p* = .018, Cohen’s d = 0.10) and “I can talk to my family” (mean 3.7 [SD 1.1] and versus 3.6 [SD1.1], t = 2.60, *p* = .009, Cohen’s d = 0.11). Moreover, there was a statistically significant pre- to post increase in their stating “If I sought out professional help, I would be less satisfied with myself” (mean 2.3 [SD 1.0] versus 2.4 [SD 1.1], t = -2.13, *p* = .34, Cohen’s d = − 0.09. The final statistically significant change from pre- to post-campaign in the male group was an increase in their willingness to contact their GP for suicide thoughts (37% versus 43%, Z = -2.51, *p* = .02).

### Subgroup analysis: campaign awareness

Among the 1149 participants in the post-campaign survey, 265 (23.1%, no gender difference) responded that they had noticed a campaign on suicide awareness in the region without being prompted. In the post-campaign survey, significantly fewer individuals in this group stated that they would contact someone’s next of kin if they were worried about them, and significantly fewer individuals agreed with the statement that there is a risk of evoking suicide thoughts by asking about them (Supplementary Tables 1–2).

### Subgroup analysis: attrition

Of the 1751 individuals who responded to the pre-campaign survey, 602 (34%) were lost to follow-up/the post-campaign survey as they did not respond to the invitation to participate. When comparing the baseline responses of the attrition group and the retention group, the following statistically significant differences were found: There were more men (54.5% versus 48.7%, X^2^ = 5.24, *p* = .024), more individuals < 30 years of age (31.0% versus 14.1%, X^2^ = 90.93, *p* < .001), fewer individuals in the group with higher education (MA degrees) (20.6% versus 28.0%, X^2^ = 20.91, *p* > .001), and fewer individuals working in the private sector (35.9% versus 42.3%, X^2^ = 14.46, *p* = .001) in the attrition group. Moreover, in the attrition group, more individuals agreed with the statements “If I sought out professional help I would be less satisfied with myself” (t = 2.35, *p* = .019), “I would feel inferior if I asked a mental help counselor for help” (t = 2.15, *p* = .031), and “I would have negative feelings for myself if I couldn’t solve my own problems” (t = 2.79, *p* = .005). Finally, more individuals in the attrition group disagreed with the statements “My self-esteem would NOT be threatened if I sought professional help” (t -2.30, *p* = .021) and “I would feel OK deciding to seek out professional help” (t -2.95, *p* = .003).

## Discussion

This is the first Norwegian regional population study of potential effects on attitudes to suicide, attitudes to mental problems, and help-seeking after a suicide prevention media campaign utilizing print, digital, and social media channels. In summary, there were very few and very small statistically significant effects of the campaign. Moreover, the few significant effects that were found were indicative of both increases and decreases in desirable attitudes to suicide, mental illness, and help-seeking. In the subgroup who explicitly stated that they had noticed the campaign, fewer individuals would reach out to a friend with suicide thoughts compared to before the campaign. However, fewer of them agreed with the myth that there is a risk of evoking suicide thoughts if asking about them.

In the entire group of participants, and when controlling for age, fewer individuals would contact the next of kin if they were worried about a friend with suicide thoughts, and more individuals would contact their GP for their own suicide thoughts. This latter change seems to be driven by male status, as the subsequent analysis of the male participants only confirmed that significantly more of them would seek out their GP if they had suicide thoughts. Moreover, in the post-survey responses of the male group, significantly fewer reported that they could trust their friends when something goes wrong and that they could talk to their family members. In addition, significantly more of the male participants stated that they would be less satisfied with themselves of they sought professional help for psychological problems. These results, albeit few, indicate that the average male faces particular challenges in reaching out to others for help, both privately and professionally, and that this type of media campaign has not been able to improve their situation. Men with suicide thoughts or emotional problems may benefit more from campaigns using role models to convey information, practical assistance with recognizing symptoms, and active problem-solving tasks [20]. Designing campaigns that take advantage of traditional masculine norms of direct action and practical problem-solving could help men manage emotional problems and suicide thoughts in a better way.

According to the systematic review by Torok et al., small or negligible effects of public awareness campaigns on beliefs about and attitudes to suicide, mental illness, and help-seeking seem to be quite common [12]. A major problem in assessing outcomes of studies of the effect of media campaigns is the divergent methodological approaches of relevant studies [7]. Very few studies have used our method of comparing the pre-post responses of *the same sample* with a large enough N to detect statistically significant changes. A Dutch campaign that was evaluated in a population study similar to ours managed to raise awareness of a national helpline for people with suicidal ideation, but otherwise neither the overall analyses nor analyses of subgroups based on campaign awareness and visibility yielded the desired effects [8]. Both our study and that of van der Burgt et al. [8] assessed the responses of the sample a few weeks after the campaign, when any impact is likely to be greatest. It is, however, possible that respondents had insufficient time to reflect upon the campaign topics to alter their attitudes. Participating in this study may have set thoughts and deliberations in motion that may become evident in beneficial, help-seeking actions further ahead, e.g., if the respondents should experience suicide thoughts or mental health problems. It is also possible that altering attitudes requires repeated and consistent public information efforts over a longer time span. We have assessed attitudes only, and have no information on possible effects of the campaign on actual behavioral changes in the population of the Mid-Norway region, such as increased number of patients contacting health services for suicidal ideation or mental health problems. Nor do we know whether the campaign has had any effect on rates of self-harm or suicide in the population of this region. Although changes in behavior are unlikely to occur in the absence of changes in attitudes, there is a remote possibility that individuals who experience mental ill health or suicide thoughts would seek out help in such situations even if they had previously stated their non-interest in doing so.

It is also important to bear in mind that our results represent average responses and that responses from individuals and subgroups within the sample will vary in both desirable and undesirable directions. For some subgroups, media campaign may have detrimental effects. It has been reported that individuals experiencing depressive symptoms may perceive a campaign intended to encourage help-seeking as more negative than individuals without depressive symptoms [21]. Increasing levels of self-stigma in this subgroup may account for this phenomenon [22]. Moreover, self-stigma seems to interact with gender. In men with depression, stigmatizing attitudes towards themselves and their own help-seeking may be higher than in women with depression [23]. It is a limitation of the present study that we did not ask the participants about their own mental health.

Some of the contents and strategies of the media campaign itself could also account for our lack of convincing significant effects. We employed several different means of exposure, as well as several different messages, such as direct information about where to seek help for suicide thoughts, interviews with survivors of suicide attempts, and appeals to get help from individuals who had lost family members to suicide. Moreover, the main way of presenting the campaign was through a one-minute film clip of a man shown in different life situations, where the message *Talk about suicide thoughts* was displayed only at the end. Effective public awareness campaigns for depression treatment and suicide prevention are associated with few, short, and specific messages that are repeatedly exposed [24]. The low click-through rates (all < 1%) indicates that at least the digital campaign material failed to engage with the exposed group. Possibly, our campaign was too complex and the message not clear or specific enough to evoke the interest of the recipients and to have an effect on their attitudes.

The result of the attrition analysis indicate that the group lost to follow-up (34% of the original sample) consisted of individuals who had more stigmatizing attitudes to suicide, mental illness, and help-seeking for such problems. These characteristics are among those a media suicide prevention campaign is intended to change. If these individuals had participated in the post-campaign survey, it is possible that the impact of the campaign would have been different.

Moreover, the timing of the campaign at right before Christmas may have been unfortunate. With increases in advertisements for gifts and festival events as well as this being a generally busy time of the year for most people, the campaign messages may have drowned in a very cluttered media landscape.

A final suggested explanation for our lack of effects was that the awareness campaign took place immediately after the Covid-19 pandemic. During the pandemic, numerous media outlets described and discussed reports of people suffering from loneliness, worry, and psychological distress due to physical and social isolation. In particular, the negative effects of the pandemic on individuals who already struggled psychologically (e.g., people receiving mental health care) may have raised the awareness of psychological problems and the importance of reaching out, negating any additional benefits of the suicide prevention campaign to the mental health literacy of the population.

### Strengths and limitations

A major strength of our study is our pre-post comparison of the same sample. Our sample was also large enough to facilitate subgroup and covariance analyses. In addition, we assessed beliefs and attitudes with multiple items and questions, increasing the possibility of detecting important changes.

Apart from the fact that the campaign material may have been too general and unclear (discussed above), a major limitation is our low campaign awareness rate at 23%. However, this proportion is much higher than the 11–15% visibility rate of van der Burgt et al. [8]. In a similar vein, our response rate was low, with only 23% of those initially contacted agreeing to participate in the baseline survey. Again, this number is higher than other studies of baseline attitude measurements, such as 12% in Australia [11] but considerably lower than 71% in Singapore [25].

A further limitation is that this was not a randomized controlled trial, so it is difficult to ascribe causality to any of the effects. Moreover, we had no information on experiences with mental or somatic illness, suicide thoughts, or help-seeking in our respondents. Such experiences may influence attitudes to these topics as well as willingness to participate in this type of research. In addition, we had no information on the participants’ ethnic or minority background, factors that are known to affect beliefs and attitudes to suicide and help-seeking [11]. Finally, although our overall N was high, we did not have sufficient power to analyze the effects of each campaign element separately in the small group that was aware of the campaign.

## Conclusion and practical implications

In this first Norwegian population-based study of a suicide prevention media campaign, there were only marginal significant effects on beliefs about and attitudes to suicide, mental illness, and help-seeking. This finding will inform our next efforts in several ways in our aim to design campaigns that are more successful in terms of visibility and effect. Importantly, the following measures are merely suggestions for improvement, and we have no evidence that they will have an impact in this particular context. First, we will aim to design campaign messages that are simpler and clearer. Second, we will analyze this data set from the Mid-Norway region in combination with the data sets from future regional campaigns with more advanced statistical methods aiming to identify response clusters that indicate subgroups in need of targeted interventions, e.g., male respondents with few social contacts.

Third, the next campaigns planned for other regions of Norway will employ a theory-driven approach. When studying human behavior, it is generally recommended to use theories that encompass both attitudes and intentions that determine people’s actions [26]. Interventions based on The Theory of Planned Behavior [27] has been shown to explain 40–60% of the intention to seek help for mental health issues [28, 29]. In the future, we will let this theory inform both the design and evaluation of our awareness campaigns.

In summary, this study adds to previous reports of media campaigns having small and short-lived effects on attitudes to suicide and help-seeking for suicide thoughts [12, 30]. In general, public information campaigns intended to foster healthy behaviour in the entire population seem to have modest positive effects at best [31]. Significant and lasting changes in attitudes to suicide and help-seeking could perhaps be better generated by increasing efforts to help individuals at known risk for suicide [30] of by local initiatives, such as training primary health care workers in the detection and treatment of suicide thoughts [32].

### Electronic supplementary material

Below is the link to the electronic supplementary material.

Supplementary Material 1

## Data Availability

The data that support the findings of this study are available from Norstat but restrictions apply to the availability of these data, which were used under license for the current study, and so are not publicly available. Data are however available from the authors upon reasonable request and with permission of Norstat.
