# Supplementary material for: Change in attitudes after a suicide prevention media campaign in the Mid-Norway region
Source: BMC Psychiatry. 2024 Jun 14;24:444. doi: 10.1186/s12888-024-05905-x (PMC11177419; doi:10.1186/s12888-024-05905-x)
Supplement: Supplementary file 1 — Supplementary Material 1 [file 12888_2024_5905_MOESM1_ESM.docx]

Supplementary Table 1. Attitudes to suicide and help-seeking pre and post campaign in Mid-Norway in those who had seen the campaign (N=265)

|  | **Pre**  **Mean (SD)** | **Post**  **Mean (SD)** | **t-test** |
| --- | --- | --- | --- |
| **Attitudes to suicide** |  |  |  |
| I am prepared to help a suicidal person by contacting/talking to him/her | 4.3 (0.9) | 4.1 (1.0) | 2.43, p = .016 * |
| When a person has decided to take their life, it can’t be prevented | 2.1 (1.1) | 2.1 (1.1) | -0.12, p = .905 |
| Suicide is one’s own business that others should not interfere with | 1.6 (0.8) | 1.6 (0.8) | 0.09, p = .928 |
| There is a risk of evoking suicide thoughts if one asks about it | 2.6 (1.0) | 2.5 (1.0) | 2.17, p = .031 * |
| Suicide is a topic that should not be discussed | 1.7 (0.9) | 1.7 (1.0) | -1.08, p = .280 |
| Suicide occurs without warning | 3.0 (1.1) | 2.9 (1.1) | 0.82, p = .415 |
| If I had suicide thoughts, I am confident of getting good health care | 3.1 (1.1) | 3.1 (1.2) | -0.74, p = .461 |
|  |  |  |  |
| **Attitudes to help seeking** |  |  |  |
| If I had depression, I would not tell anyone | 2.8 (1.2) | 2.8 (1.1) | 0.49, p = .350 |
| If I were experiencing a serious emotional crisis, I am confident that I would get good professional help | 2.7 (0.9) | 2.7 (0.9) | -0.17, p = .868 |
| People should work out their own problems, getting psychological help should be the last resort | 1.5 (0.7) | 1.5 (0.8) | -0.43, p = .668 |
| Emotional problems, like many things, tend to work out by themselves | 2.4 (0.8) | 2.4 (0.8) | -0.22, p = .824 |
|  |  |  |  |
| **Self-stigma towards help seeking** |  |  |  |
| If I sought professional help I would be less satisfied with myself | 2.1 (1.0) | 2.2 (1.0) | -1.37, p = .170 |
| My self-esteem would NOT be threatened if I sought professional help | 4.0 (1.1) | 3.9 (1.1) | 1.07, p = .288 |
| I would feel inferior if I asked a mental help counselor for help | 2.1 (1.0) | 2.1 (1.1) | 0.00, p = 1.000 |
| I would have negative feelings for myself if I couldn’t solve my own problems | 2.8 (1.1) | 2.7 (1.1) | 0.11, p = .915 |
| I would feel OK deciding to seek out professional help | 4.1 (1.0) | 4.1 (1.0) | 0.98, p = .329 |
|  |  |  |  |
| **Social support** |  |  |  |
| If I needed it, I would feel OK seeking help from family and friends for emotional problems | 3.8 (1.0) | 3.6 (1.0) | 1.97, p = .050 |
| I get the emotional support I need from my family | 3.7 (1.1) | 3.6 (1.0) | 1.75, p = .082 |
| I can trust my friends when something goes wrong | 3.8 (1.0) | 3.6 (1.1) | 1.77, p = .078 |
| I can talk to my family about my problems | 3.7 (1.1) | 3.6 (1.1) | 1.33, p = .186 |
| I can talk to my friends about my problems | 3.6 (1.1) | 3.6 (1.1) | 0.46, p = .645 |

Paired samples t-test, *: p <.05.

Supplementary Table 2. Attitudes to help-seeking alternatives pre and post campaign in Mid-Norway in those who had seen the campaign (N=265)

|  | **Pre**  **N (%)** | **Post**  **N (%)** | **X^2^ -test** |
| --- | --- | --- | --- |
| **What would you do if you were worried about a suicidal friend?** |  |  |  |
| I would talk to that person about it | 167 (63.0) | 167 (63.0) | 0.00, p = 1.000 |
| I would ask him/her to contact professional help | 137 (51.7) | 138 (52.1) | 0.00, p = 1.000 |
| I would contact his/her next-of-kin | 124 (46.8) | 106 (40.0) | 3.14, p = .076 |
| I would contact health care services myself | 93 (35.1) | 77 (29.1) | 2.96, p = .085 |
| I am not sure what I would have done | 37 (14.0) | 34 (12.8) | 0.11, p = .735 |
|  |  |  |  |
| **If you had suicide thoughts, where would you seek help?** |  |  |  |
| Family/partner/spouse/significant other | 103 (38.9) | 107 (40.4) | 0.15, p = .703 |
| Friends/colleagues | 58 (21.9) | 44 (16.6) | 3.25, p = .071 |
| GP | 106 (40.0) | 113 (42.6) | 0.46, p = .500 |
| Emergency services | 34 (12.8) | 37 (14.0) | 0.11, p = .742 |
| Local mental health services | 67 (25.3) | 79 (29.8) | 1,78, p = .182 |
| Telephone help line /emergency hotline | 78 (29.4) | 77 (29.1) | 0.00, p = 1.000 |
| Internet | 28 (10.6) | 38 (14.3) | 1.84, p = .175 |
| I would not seek help or information | 19 (7.2) | 22 (8.3) | 0.11, p = .607 |
| I don’t know where I would seek help | 39 (14.7) | 27 (10.2) | 3.56, p = .059 |

Two-related samples McNemar test.
